# Self-Bending Behavior and Varying Bending Stiffness of Black Phosphorus/Molybdenum Disulfide (BP/MoS_2_) Heterostructure

**DOI:** 10.3390/nano12203635

**Published:** 2022-10-17

**Authors:** Dong Li, Yonggang Zheng, Hongwu Zhang, Hongfei Ye

**Affiliations:** International Research Center for Computational Mechanics, State Key Laboratory of Structural Analysis for Industrial Equipment, Department of Engineering Mechanics, Faculty of Vehicle Engineering and Mechanics, Dalian University of Technology, Dalian 116024, China

**Keywords:** black phosphorus, molybdenum disulfide, heterostructure, bending stiffness, self-bending behavior, molecular dynamics

## Abstract

Vertically-stacked black phosphorus/molybdenum disulfide (BP/MoS_2_) heterostructures have broad prospects in flexible electronics. Bending is a common and highly concerned deformation for these flexible devices. However, the discrepancy in structures and properties among the components of 2D heterostructures often induces complex bending deformations. Here, the bending behaviors of BP, MoS_2_ and BP/MoS_2_ are investigated based on a molecular dynamics simulation. Compared with the constant bending stiffness of individual BP and MoS_2_, that of BP/MoS_2_ varies with the bending angle. Notably, a self-bending configuration induced by the lattice mismatch and size difference is found in BP/MoS_2_. The corresponding self-bending amplitude depends on the degree of size difference of each component and the “soft/hard” competition between them. Moreover, the size difference leads to a weakened bending stiffness, which is ascribed to the reduction in interlayer interaction. A prediction formula is proposed to evaluate the bending stiffness of BP/MoS_2_ with the size difference. This finding reveals novel ways for regulating the bending properties of 2D heterostructures, including the bending angle, characteristic size and stacking order. It offers an effective strategy for designing flexible devices with tunable bending performance.

## 1. Introduction

In recent years, the number of 2D materials has been growing continuously with surprising speed, and about 5600 layered compounds have been discovered [1,2]. The large family of 2D materials can thus cover a wide range of material properties that make them attractive research objects and potential candidates for numerous applications [3,4,5,6,7]. For instance, graphene has ultrahigh mechanical strength and excellent thermal conductivity [3,8]. Black phosphorus (BP) exhibits high carrier mobility and anisotropic plane characteristics [4,9]. Molybdenum disulfide (MoS_2_) possesses excellent electrical transport properties and adjustable bandgap structure [5,10]. Notably, the novel composites formed by the vertical assembly of single-layer 2D materials (called van der Waals heterostructures) often exhibit many exciting properties and functions, which opens a new way to explore the high-performance applications based on 2D heterostructures [11,12,13]. The latest progress indicates that the heterostructure formed by the stacking of BP and MoS_2_ nanosheets shows many excellent photoelectric characteristics [14,15], which leads to a broad application prospect in flexible photodetectors and field-effect transistors [16,17].

Owing to the ultrathin and ultrasoft nature of 2D materials, they usually undergo various complex deformations in the design and application, such as bending, wrinkling, and rippling [18,19]. Therefore, it is of great significance to understand these bending properties and deformation behaviors for assessing the quality and life of relevant devices. Bending stiffness is a fundamental property for evaluating the deformation and function of flexible electronics, however, addressing how to accurately evaluate the bending stiffness is still a challenging task [20,21]. Even for the single-layer 2D materials, the bending stiffness measured by different methods has shown extremely divergent results [22,23,24]. As for the multi-layer homogeneous 2D materials, the bending stiffness depends on the number of stacking layers in the vertical direction significantly [24,25], but it may not satisfy the quantitative relation ($D\;\sim \;t^{3}$) in continuum mechanics [21]. Furthermore, the bending stiffness of multi-layer graphene depends on the bending angle. Their bending stiffness decreases with increasing bending angle due to the increase in interlayer slip, which has been confirmed in the relevant experiment [26,27]. Notably, the double-layer BP exhibits an ultrahigh and strong discontinuous bending stiffness in the bending process, which is attributed to the interfacial structural transition from commensurability-induced self-locking to unlocking [28]. Therefore, for the multi-layer heterogeneous 2D materials, their mechanical properties and deformation behaviors are usually more complex and diverse due to the accumulation of various component materials [29,30]. This may cause many challenges and uncertainties for their performance evaluation and ensuring their safe service. It has been reported that the interlayer interaction and lattice mismatch of 2D heterostructures often induces significant changes in structural morphology and mechanical property [31,32], e.g., a buckled atomic structure in the graphene/hBN heterostructure resulted from a small lattice mismatch [31]. So far, however, few studies have focused on the bending properties and the corresponding mechanisms of 2D heterostructures, especially for the bending behavior of BP/MoS_2_ heterostructures.

In this work, a computational method is developed for predicting the bidirectional bending behavior of 2D heterostructures. Based on the molecular dynamics (MD) simulations, the bending properties of BP, MoS_2_ and BP/MoS_2_ are investigated, and then the self-bending behavior and varying bending stiffness are found in BP/MoS_2_. Furthermore, we illuminate the evolution of self-bending behavior and the mechanism of varying bending stiffness by analyzing the bilayer components and interlayer interaction. A prediction formula is proposed to assess the bending stiffness of BP/MoS_2_ under the size differences. These results provide a vital basis for fabricating flexible devices with adjustable bending properties based on a 2D heterostructure.

## 2. Models and Methodology

### 2.1. Numerical Models

**Figure 1:**
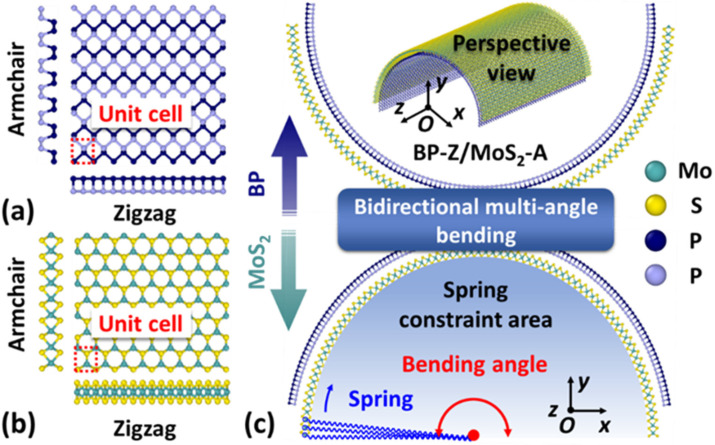
(**a**) and (**b**) show the molecular models of SLBP and SLMoS_2_, respectively. The structures in the red dotted box are the representative unit cells selected in this paper; (**c**) illustrates the bidirectional bending models of BP-Z/MoS_2_-A (bending angle is 180°) formed by the stacking of BP-Z and MoS_2_-A. The green and blue arrows represent bending towards MoS_2_ and BP, respectively. The inset is the perspective view of BP-Z/MoS_2_-A with a cylindrical shell structure. The light blue section is the traction area consisting of coaxial springs. The blue broken lines represent springs. The red point is the central axis of curvature of the cylindrical shell structure.

Single-layer BP (SLBP) is a puckered molecule composed of two layers of phosphorus (P) atoms. Each P atom is connected to three neighboring P atoms, as shown in Figure 1a. Single-layer MoS_2_ (SLMoS_2_) with a honeycomb-like structure is formed by three layers of atoms. The Mo atomic layer is sandwiched between the two S atomic layers, as depicted in Figure 1b. According to the geometric characteristics of the two boundaries of SLBP and SLMoS_2_, the chiral directions can be divided into armchair (A) and zigzag (Z) types. There are obvious differences in the mechanical properties along different chiral directions [33,34,35]. BP/MoS_2_ heterostructure is vertically stacked by SLBP and SLMoS_2_ nanosheets under the van der Waals interaction. By means of the chiral directions, BP/MoS_2_ with four types of stacking orders along the bending direction are established, namely BP-A/MoS_2_-A, BP-Z/MoS_2_-A, BP-A/MoS_2_-Z, BP-Z/MoS_2_-Z. Figure 1c shows the computational model of bidirectional multi-angle bending of BP-Z/MoS_2_-A, which is formed by BP and MoS_2_ components with chiral directions of Z and A at the bending boundary, respectively. The bidirectional multi-angle bending means that BP/MoS_2_ is bent to multiple angles towards the two directions of BP and MoS_2_. Here, the BP-Z/MoS_2_-A with two bending angles of 180° is depicted in Figure 1c. The characteristic sizes of the computational models are about 200 Å × 200 Å, and the detailed characteristic sizes are listed in Table 1. Due to the different lattice constants (lattice mismatch) of BP and MoS_2_ [36,37], the characteristic sizes of bilayer components of BP/MoS_2_ are difficult to keep consistent and often exhibit a small size difference. The size difference is defined as $SD=\left(L_{\mathrm{BP}}-L_{{\mathrm{MoS}}_{2}}\right)/L_{{\mathrm{MoS}}_{2}}$, $L_{\mathrm{BP}}$ and $L_{{\mathrm{MoS}}_{2}}$ are the lengths of SLBP and SLMoS_2_ along the bending boundary, respectively.

### 2.2. Computational Method

Based on the continuum mechanics theory, the bending stiffness can be calculated by the following equation [34,38],

(1) $$\frac{1}{2}D\kappa^{2}=∆U$$

where, D is bending stiffness, κ is curvature of 2D materials, $∆U=U-U_{0}$ is the strain energy density. U and $U_{0}$ correspond to the potential energy density of the curved and undeformed configurations, respectively. For single-layer 2D materials, the undeformed flat structure generally possesses the minimum potential energy relative to the bending configuration. For 2D heterostructures, however, the van der Waals force induces the reconstruction of interlayer structure at the boundary region due to lattice mismatch and size difference, making them difficult to keep a flat structure at the natural (unconstrained) state. Thus, Equation (1) is no longer applicable when the configuration of minimum potential energy is not a flat structure, which can be improved to calculate the bending stiffness of 2D heterostructures as,

(2) $$\frac{1}{2}D{\left(\kappa -\kappa_{\min}\right)}^{2}=U-U_{\min}$$

where, $\kappa_{\min}$ and $U_{\min}$ are the curvature and potential energy density of the configuration with minimum potential energy, respectively. In fact, $\kappa_{\min}$ and $U_{\min}$ are always unknown for 2D heterostructures. They are difficult to be accurately determined by the numerical simulations. Here, based on the proposed bidirectional multi-angle bending, $\kappa_{\min}$, $U_{\min}$ and D can be well predicted by the quadratic fitting of the ∆U−κ relationship. It can effectively determine the corresponding configuration with minimum potential energy.

The bending loading is applied based on the spring-driven method. The cylindrical shell configurations of SLBP, SLMoS_2_ and BP/MoS_2_ are first established through geometric mapping, as shown in Figure 1c. Then, the appointed atoms are constrained to the mapped circumference (curvature radius) through a group of coaxial springs with high stiffness ($K=20\;\mathrm{eV}/{Å}^{2}$). One end of the springs is connected to the appointed atoms, and the other end is fixed to the central axis of curvature of the cylindrical shell configuration. For SLBP, the curvature radius of one layer of P atoms is restrained by the springs, while the other layer can adjust freely. For SLMoS_2_, the middle Mo atomic layer is constrained, but the upper and lower S atomic layers are free to self-adjust. As for BP/MoS_2_ stacked by SLBP and SLMoS_2_ with different characteristic sizes, only the large-size material is restrained, and the small-size material can be adsorbed on the surface of the constrained material under the van der Waals interaction. This constraint mode has only an out-of-plane force along the radial direction and allows structural adjustments in the bending plane. In order to improve the computational accuracy and systematically investigate the bending behaviors of SLBP, SLMoS_2_ and BP/MoS_2_, the simulation models with multiple bending angles are established under the same characteristic size. Here, one type of model is built every 10° between 0 and 90° and every 30° between 90° and 180°.

### 2.3. Simulation Details

MD simulations are performed in the LAMMPS package, and the OVITO software is used to visualize the molecular configurations. The Stillinger-Weber potential is employed here to describe the covalent interaction of BP and MoS_2_ atoms [39,40]. It is widely used to characterize the mechanical properties, and the effectiveness in describing the bending properties has been demonstrated in previous studies [34,41]. The interaction between BP and MoS_2_ molecules is calculated by the Lennard-Jones (LJ) potential with a cut-off distance of 12 Å. The formula of 12-6 LJ potential function is $U\left(r\right)=4\varepsilon \left[{\left(\sigma /r\right)}^{12}-{\left(\sigma /r\right)}^{6}\right]$, where, r is the distance between the atoms of BP and MoS_2_ molecules. The corresponding parameters σ and ε are calculated according to the Lorentz-Berthelot mixing rules [42], as depicted in Table 2. The simulations are carried out under the NVT ensemble with the time step of 1 fs. The Nosé-Hoover thermostat is utilized to control the system temperature at 0.01 K. The computational results are statistically analyzed when the system energy is in a steady state.

## 3. Results and Discussions

### 3.1. Bending Stiffness

**Figure 2:**
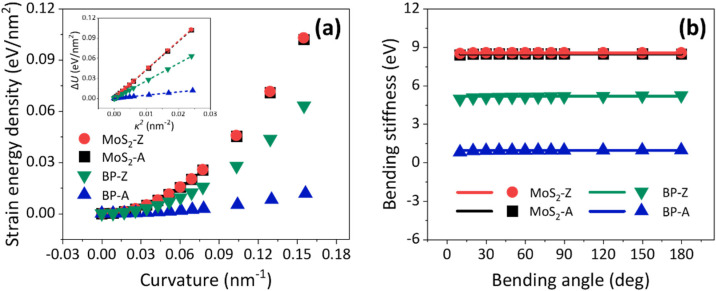
(**a**) The relationships between the strain energy density and curvature of SLBP and SLMoS_2_. The inset shows the relation between the strain energy density (∆U) and curvature square ($\kappa^{2}$). The dotted lines are the fitting lines. (**b**) The variations of bending stiffnesses of SLBP and SLMoS_2_ with the bending angles. The scatters and solid lines are the computational results of two-point and multi-point fittings, respectively.

The bending properties of SLBP and SLMoS_2_ are examined on the basis of MD simulations. Figure 2a shows the relationship between the strain energy density and curvature. According to Equation (1), the bending stiffness of SLBP and SLMoS_2_ can be obtained by linear regression of the relation between the strain energy density and curvature square ($∆U-\kappa^{2}$), as depicted in the inset of Figure 2a. Here, taking the flat structure as the configuration of minimum potential energy, two-point and multi-point fittings are utilized to calculate the bending stiffness. Firstly, the linear regression is carried out through the two numerical samples from the flat and curved models (two-point fitting), which could reflect the change in bending stiffness with bending angle. The bending stiffness of SLBP and SLMoS_2_ is relatively stable and almost insensitive to the bending angle, as shown by the scatters in Figure 2b. Then, the bending stiffness is calculated by fitting 12 numerical samples from 0 to 180° (multi-point fitting), and the corresponding results are depicted as the solid lines in Figure 2b. The correlation coefficients of the multi-point fitting results approach 1.0. In general, the multi-point fitting can consider more samples and thus possesses higher computational accuracy and stability relative to the two-point fitting. The bending stiffnesses of SLMoS_2_ bent along the A and Z boundaries are 8.48 eV (MoS_2_-A) and 8.57 eV (MoS_2_-Z), respectively. It is indicated that their bending stiffness almost does not depend on the chiral directions. As for SLBP, however, the bending stiffnesses of BP-A and BP-Z are 0.96 eV and 5.20 eV, respectively. Their bending stiffness varies more than five times in different chiral directions, which exhibits a significant chiral dependence.

**Figure 3:**
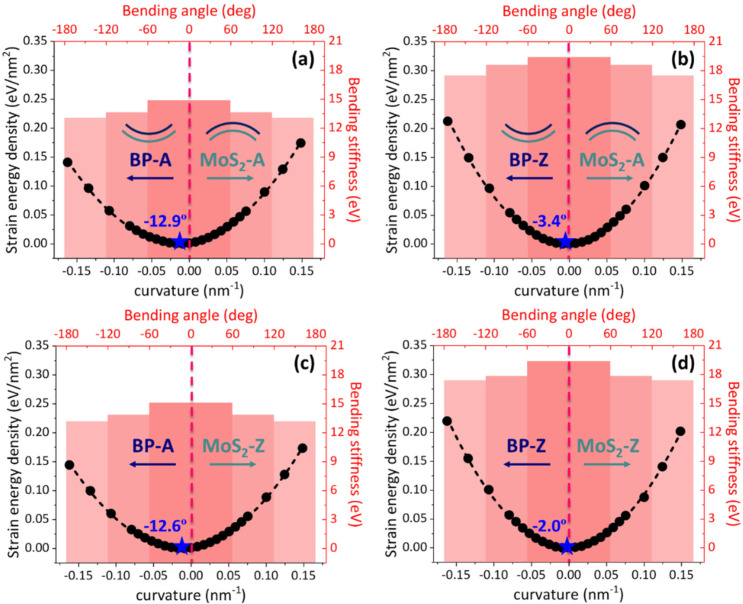
The relationships between the strain energy density and bending stiffness of BP/MoS_2_ with four stacking orders versus the curvature (black axis) and bending angle (red axis), respectively. (**a**) BP-A/MoS_2_-A. (**b**) BP-Z/MoS_2_-A. (**c**) BP-A/MoS_2_-Z. (**d**) BP-Z/MoS_2_-Z. The black scatters represent the relation between the strain energy density and curvature, and the black dotted line is the fitting line. The red histograms show the relationship between the bending stiffness and the three ranges of bending angles.

For BP/MoS_2_, the bidirectional bending behaviors under multiple bending angles are investigated in this section. Based on MD simulations, the relationship between the strain energy density and curvature is shown by the black scatters in Figure 3. According to Equation (2), the bending stiffnesses of BP/MoS_2_ within three ranges of bending angles can be obtained by piecewise quadratic regression of the relationship between the strain energy density and curvature (∆U−κ, the ranges of piecewise fitting are −60°~60°, −120°~120°, −180°~180°). All correlation coefficients of fitting results are larger than 0.997. Here, the bending angle and curvature are defined as positive and negative values when BP/MoS_2_ is bent towards MoS_2_ and BP, respectively. The three groups of bending stiffnesses within different ranges of bending angle are depicted in the red histograms with a gradient color. The computational results indicate that the bending stiffness of BP/MoS_2_ decreases with the increase in bending angle when they are bent to both sides of the bilayer components (the more bent, the softer). For BP-A/MoS_2_-A and BP-Z/MoS_2_-A, the bending stiffnesses reduce from 14.90 eV to 13.06 eV by 14.09% and from 19.41 eV to 17.49 eV by 10.98%, respectively. As for BP-A/MoS_2_-Z and BP-Z/MoS_2_-Z, the bending stiffnesses decrease from 15.12 eV to 13.18 eV by 14.72% and from 19.38 eV to 17.38 eV by 11.51%. Compared with the considerable and sudden reduction in bending stiffness of the double-layer BP induced by the interfacial structural transition [28], the small and gradual decreasing trend in BP /MoS_2_ is ascribed to the shrinking overlapped stacking regions between the two layers with the increase in bending angle. Notably, the bending angle-dependent bending stiffness of 2D heterostructure has also been confirmed by combining theoretical and experimental methods [26,45]. Moreover, since the difference in bending stiffness of MoS_2_ with different chiral directions is not obvious, the discrepancy in bending stiffness of BP/MoS_2_ under different stacking orders mainly comes from BP. Compared with individual BP and MoS_2_, the stacking of two layers can enhance the bending stiffness of BP/MoS_2_. However, the enhanced bending stiffness is significantly smaller than that estimated by the continuum mechanics ($D\;\sim \;t^{3}$). It is because the bilayer components of BP/MoS_2_ are not connected by strong coupling interactions, but are only held together through the weak van der Waals forces.

It is worth noting that for BP/MoS_2_ heterostructure, the bidirectional bending does not start from the flat structure. It is because the natural configuration with minimum potential energy always presents a certain bending angle, which is the self-bending configuration induced by the size difference (see Table 1). Based on the relationship of ∆U−κ, the self-bending angles (amplitudes) of BP-A/MoS_2_-A, BP-Z/MoS_2_-A, BP-A/MoS_2_-Z and BP-Z/MoS_2_-Z predicted by Equation (2) are 12.9°, 3.4°, 12.6° and 2.0°, respectively. These results demonstrate that although the size difference between the bilayer components is rather small, it still causes an obvious self-bending morphology. Furthermore, it can be seen that the stacking orders of bilayer components have a notable effect on the self-bending behavior of BP/MoS_2_.

### 3.2. Size Difference-Induced Self-Bending Behavior

**Figure 4:**
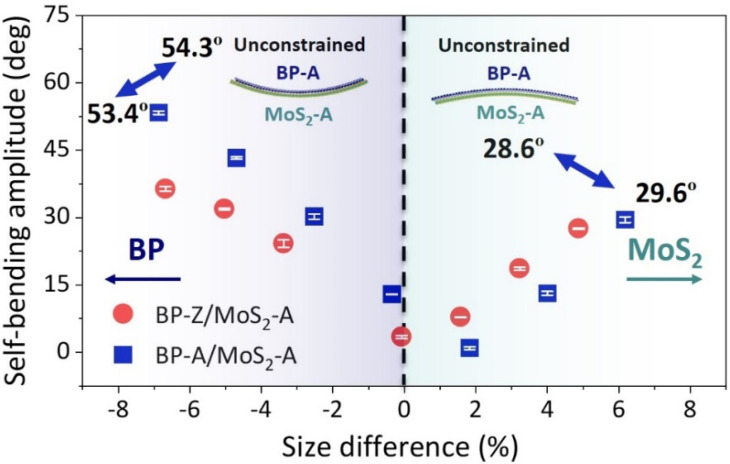
The self-bending amplitudes of BP-A/MoS_2_-A and BP-Z/MoS_2_-A with the size difference and stacking order. The errors are the average of three fitting results within different ranges around the configuration with minimum potential energy. The blue and green arrows represent the directions of self-bending towards BP and MoS_2_, respectively. The insets show the self-bending configurations of BP-A/MoS_2_-A with *SD* = −6.87% and *SD* = 6.17% without the spring constraint.

To further investigate the self-bending behavior, the variations of the self-bending amplitude of BP/MoS_2_ with the size difference and stacking order are examined as depicted in Figure 4. Compared with BP, the bending properties of MoS_2_ along the two boundaries are almost identical (see Figure 2b). Therefore, along the bending direction, the combinations of MoS_2_ of armchair boundary (A) with BP of two chiral directions (A and Z) are chosen for examination, i.e., BP-A/MoS_2_-A and BP-Z/MoS_2_-A. Here, the characteristic size of MoS_2_ keeps constant (see Table 1), while that of BP varies in the bending boundary but remains unchanged in the non-bending boundary. The magnitude of the size change is an integer multiple of the unit cell shown in Figure 1. For BP/MoS_2_, when the size of BP is larger than MoS_2_ ($L_{\mathrm{BP}}>L_{{\mathrm{MoS}}_{2}}$), the size difference is positive, otherwise it is negative.

It can be seen from Figure 4 that BP/MoS_2_ generally self-bends from a large-size material to small-size material, and the self-bending amplitude increases with the degree of size difference of the bilayer components. However, compared with $L_{\mathrm{BP}}>L_{{\mathrm{MoS}}_{2}}$ in bilayer components of BP/MoS_2_, $L_{{\mathrm{MoS}}_{2}}>L_{\mathrm{BP}}$ induces a more significant self-bending amplitude under a comparable degree of size difference. For example, the self-bending amplitudes of BP-A/MoS_2_-A towards BP and MoS_2_ are 53.4° and 29.6° when the size differences are *SD* = −6.87% and *SD* = 6.17%, respectively. This is because the bending stiffness of BP-A (0.96 eV), acting like a bending resistance, is obviously weaker than that of MoS_2_-A (8.48 eV). Thus, the bending deformation can be driven more easily by the self-adaptive adjustment under the van der Waals interaction. As for BP-Z/MoS_2_-A (5.20 eV/8.48 eV), owing to the small difference in the bending stiffness of the bilayer components, the self-bending amplitudes with comparable size differences are very close. Furthermore, the insets of Figure 4 show the two self-bending configurations of BP-A/MoS_2_-A with *SD* = −6.87% and *SD* = 6.17% without the spring constraint, which are nearly perfect cylindrical shell structures induced by the size difference. The corresponding self-bending angles are 54.3° and 28.6°. The good agreement with the predicted results based on Equation (2) verifies the validity of the proposed quadratic fitting method for calculating the self-bending amplitude. The above results reveal that the self-bending amplitude of BP/MoS_2_ is closely related to the size difference of the component materials and is significantly dependent on the “soft/hard” competition between the bilayer components.

### 3.3. Size Difference-Induced Weakened Bending Stiffness

**Figure 5:**
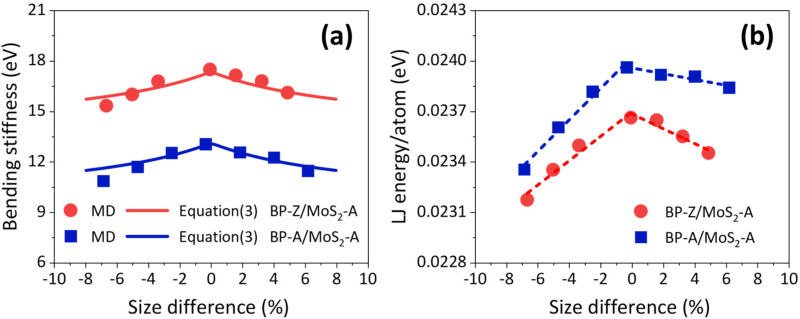
(**a**) The variations of bending stiffnesses of BP-A/MoS_2_-A and BP-Z/MoS_2_-A with the size differences. Here, the scatters represent the computational results of MD simulations. The solid lines are the predicted results based on Equation (3). (**b**) The relationships between the LJ energy/atom and size difference. The dotted lines are the fitting lines.

The size difference between the bilayer components of BP/MoS_2_ not only induces the self-bending behavior but also has a considerable effect on the bending stiffness. The bending stiffness of BP/MoS_2_ in the subsequent discussion is based on the results of the quadratic fitting with −180°~180° numerical samples. Figure 5a shows the variations of the bending stiffnesses of BP-A/MoS_2_-A and BP-Z/MoS_2_-A with the size differences. It can be seen that both the increase and decrease in the characteristic size of BP lead to a reduction in the bending stiffness of BP/MoS_2_. For BP-A/MoS_2_-A, the bending stiffness decreases from 13.06 eV to 10.86 eV and 11.48 eV when the size difference changes from the minimum case (*SD* ≈ 0) to *SD* = −6.87% and *SD* = 6.17%, respectively. As for BP-Z/MoS_2_-A, the bending stiffness reduces from 17.49 eV to 15.33 eV and 16.11 eV when the size difference reaches *SD* = −6.68% and *SD* = 4.88%, respectively. In order to elucidate the reasons for the size difference-induced weakened bending stiffness of BP/MoS_2_, the average LJ energies of each atom of BP-A/MoS_2_-A and BP-Z/MoS_2_-A under different bending amplitudes are calculated, as depicted in Figure 5b. It is found that the LJ energy of each atom decreases with the increase in the size difference. Notably, the LJ energy/atom decreases more significantly for the negative size difference. It implies that the reduction in the characteristic size of components is more likely to weaken the bending stiffness. The above results illuminate that the LJ energy plays the adhesive role in 2D heterostructures, however, the size difference of components induces the decrease in interlayer adhesion, which results in a weakening of bending performance of BP/MoS_2_.

### 3.4. Prediction Formula

Constructing an empirical formula for predicting the bending stiffness of BP/MoS_2_ with different characteristic sizes and size differences is of great significance, which greatly facilitates the examination of bending properties in related high-performance flexible devices. It can be seen from the above results that the contribution of bending stiffnesses of BP/MoS_2_ comes from the bilayer components and interlayer interaction. However, the size difference between the components usually causes a weakening of the interlayer interaction. Thus, the prediction formula of bending stiffness is expressed,

(3) $$D_{\mathrm{pre}}=D_{\mathrm{BP}}+D_{{\mathrm{MoS}}_{2}}+D_{\mathrm{Int}}$$

where, $D_{\mathrm{pre}}$ is the predicted result of bending stiffness, $D_{\mathrm{BP}}$, $D_{{\mathrm{MoS}}_{2}}$ and $D_{\mathrm{Int}}=D_{\mathrm{Int}}^{\prime }\left(\frac{SS_{0}}{\left|SS\right|+SS_{0}}\right)$ are the bending stiffnesses of BP, MoS_2_ and interlayer interaction, respectively. Here, SS is the discrepancy in the characteristic size of bilayer components of BP/MoS_2_. $D_{\mathrm{Int}}^{\prime }=3.67\;\mathrm{eV}$ and $SS_{0}=20\;Å$ are the fitting results based on MD simulations. According to Equation (3), the bending stiffnesses of BP/MoS_2_ with four stacking orders and the minimum size difference are predicted, as shown in Table 3. The predicted results are in good agreement with MD simulations (the average error is about 0.69%). Furthermore, the predicted bending stiffnesses of BP-A/MoS_2_-A and BP-Z/MoS_2_-A with the size difference ranges of −8% to 8% are given, as depicted by the solid lines in Figure 5a. It can be found that their bending stiffness decreases with the increase in the size difference, which is ascribed to the gradual reduction in the contribution of interlayer interaction.

## 4. Conclusions

A computational method for evaluating the bidirectional bending property of 2D heterostructure is developed in this paper. The bending behaviors of SLBP, SLMoS_2_ and BP/MoS_2_ are investigated based on MD simulations. The results indicate that the bending stiffnesses of SLMoS_2_ are almost identical along the two boundaries, while that of SLBP significantly depends on the chiral directions. Compared with SLBP and SLMoS_2_, the bending stiffness of BP/MoS_2_ varies with the bending angle. As the bending angle towards two sides increases (bidirectional bending), BP/MoS_2_ exhibits the bending behavior of “the more bent, the softer”. It is worth noting that the size difference induces BP/MoS_2_ to form a self-bending configuration, and this behavior is obviously dependent on the competitions of characteristic size and bending resistance between the bilayer components. Here, the orientation of small-size and soft components is more easily driven to form the self-bending morphology. By analyzing the LJ energy of each atom, the size difference-induced weakening of bending stiffness is attributed to the decrease in interlayer interactions. Moreover, a prediction formula is proposed for evaluating the bending stiffness of BP/MoS_2_ based on the proposed mechanism. Although the present work focuses on the bending behavior of BP/MoS_2_, the relevant conclusions will shed light on the other 2D heterostructures. This finding provides a valuable strategy to regulate the bending performance of 2D heterostructure-based flexible electronics by adjusting the bending angle, characteristic size and stacking orders of each component.

## Figures and Tables

**Table 1:** The characteristic sizes of SLBP, SLMoS_2_, and BP/MoS_2_ with four types of stacking orders.

**Type**	**BP-A**	**BP-Z**	**MoS_2_-A**	**MoS_2_-Z**
Length (Å)	200.00	200.55	200.70	201.00
**Type**	**BP-A/MoS_2_-A**	**BP-Z/MoS_2_-A**	**BP-A/MoS_2_-Z**	**BP-Z/MoS_2_-Z**
*SD* (%)	−0.35	−0.07	−0.50	−0.22

**Table 2:** The LJ parameters of BP and MoS_2._

Parameters	Mo-Mo [43]	S-S [43]	P-P [44]	Mo-P	S-P
*σ* (Å)	4.20	3.13	3.695	3.9475	3.4125
*ε* (eV)	0.000586	0.01386	0.0132	0.002781	0.013526

**Table 3:** The bending stiffnesses of BP/MoS_2_ with four stacking orders.

Type	|SS| (Å)	MD (eV)	Prediction (eV)	Error (%)
BP-A/MoS_2_-A	0.70	13.06	12.99	0.54
BP-Z/MoS_2_-A	0.15	17.49	17.32	0.97
BP-A/MoS_2_-Z	1.00	13.18	12.03	1.14
BP-Z/MoS_2_-Z	0.45	17.38	17.36	0.12

## Data Availability

The data presented in this study are available on request from the corresponding author.
